# Right and left neglect are not anatomically homologous: A voxel-lesion symptom mapping study

**DOI:** 10.1016/j.neuropsychologia.2021.108024

**Published:** 2021-11-12

**Authors:** Margaret Jane Moore, Celine R. Gillebert, Nele Demeyere

**Affiliations:** a University of Oxford, Department of Experimental Psychology, Radcliffe Observatory Quarter, Oxford, OX2 6GG, United Kingdom; b Department of Brain and Cognition, KU Leuven, Tiensestraat 102 Box 3711, 3000, Leuven, Belgium

**Keywords:** Stroke, Cognitive assessment, Neglect, Spatial attention, Lesion symptom-mapping

## Abstract

Visuospatial neglect is a heterogenous syndrome which can occur following damage to either right or left hemisphere areas. This study employs voxel-lesion symptom mapping to identify the neural correlates of left and right egocentric and allocentric neglect in a large acute stroke cohort.

A cohort of 446 acute stroke survivors (age = 26–95, 44% female) completed neuropsychological neglect assessment and routine clinical imaging. Similar to previous investigations, left egocentric and left allocentric neglect were associated with damage to distinct clusters of voxels within the posterior parietal and temporo-parietal junction areas. Unlike previous investigations, right egocentric neglect was found to most strongly associated with damage to more posterior voxels within left occipital cortical areas. Right allocentric neglect was found to be most strongly associated with damage to the anterior limb of the left internal capsule. Interestingly, the right hemisphere homologues of the areas implicated in right-lateralised neglect were not overlapping with those associated with left neglect impairment. This dissociation was present across both egocentric and allocentric neglect impairment.

The results of this investigation suggest that right egocentric/allocentric neglect should not be characterised as a consequence of damage to left-hemisphere homologues of the right hemisphere attentional systems. These findings support the characterisation of visuospatial neglect as a heterogenous cluster of impairments rather than a unitary syndrome and provide novel insight into the neural correlates of spatial attention.

## Abbreviations:

FLAIR –: Fluid Attenuated Inversion Recovery
MCA –: Middle Cerebral Artery
OCS –: Oxford Cognitive Screen
PCA –: Posterior Cerebral Artery
VLSM –: Voxel-Lesion Symptom Mapping

## 1 Introduction

Visuospatial neglect is most commonly characterised as a left-lateralised spatial attentional deficit which occurs following damage to right hemisphere temporo-parietal areas (Chechlacz et al., 2012; Parton et al., 2004). Although it is typically considered more severe and long lasting following right hemisphere lesions, visuospatial neglect also occurs frequently following left hemisphere damage (Demeyere and Gillebert, 2019; Stone et al., 1992, 1993; Suchan and Karnath, 2011; Ten Brink et al., 2017). For example, Demeyere and Gillebert (2019) conducted standardised neglect testing in a large, representative acute stroke cohort and found right-lateralised visuospatial neglect in 35% of participants. In addition, neglect is a highly heterogeneous condition and can impact either body-centred (egocentric) or object-centred (allocentric) reference frames (Binder et al., 1992; Demeyere and Gillebert, 2019; Moore et al., 2019; Stone et al., 1993; Ten Brink et al., 2017; Verdon et al., 2010). It is critically important to acknowledge this behavioural heterogeneity when investigating the neural correlates of the neglect syndrome in order to gain a more detailed understanding of the anatomy of spatial attention.

Given its higher prevalence and severity, previous studies have primarily focused on identifying the neural correlates of left-lateralised egocentric neglect in patients with right hemisphere damage. Left egocentric neglect has been shown to be associated with structural damage to right hemisphere regions surrounding the sylvian fissure, such as the temporoparietal junction and right superior temporal gyrus (Chechlacz et al., 2010, 2012, 2012; Hillis, 2005; Karnath et al., 2004; Molenberghs et al., 2012; Mort et al., 2003; Vossel et al., 2011), though a wide range of lesion patterns have been implicated. For example, Mort et al. (2003) suggested that damage to the right angular gyrus and parahippocampal region was critical, and Bird et al. (2006) additionally found that the white matter tracts connecting these areas were implicated in neglect. Karnath, Himmelbach and Rorden (2002) found that damage to subcortical structures including the right putamen, pulvinar, and caudate nucleus was associated with left egocentric neglect impairment. In addition, visuospatial neglect has also been found in a number of patients with damage confined to the cerebellum (Hildebrandt et al., 2002; Kim et al., 2008; Silveri, 2001). This heterogeneity in results has led to the hypothesis that visuospatial neglect represents a disconnection syndrome (Bartolomeo et al., 2007) and this claim has been supported by studies linking left visuospatial neglect to damage to the right superior longitudinal (He et al., 2007; Thiebaut de Schotten et al., 2008), inferior longitudinal (Bird, 2006), and inferior fronto-occipital fasciculi (Urbanski et al., 2008). A portion of the varied findings may also be explained by differential study populations, lesion coverage, neglect tests used and time between stroke and test. In addition, treating visuospatial neglect as a heterogenous syndrome rather than a unitary behavioural deficit may help disentangle some of these findings.

Previous lesion mapping investigations which have considered egocentric and allocentric neglect as independent constructs have consistently identified distinct neural correlates associated with each of these deficits (Chechlacz et al., 2010, 2012, 2012; Medina et al., 2008, 2008, 2008; Molenberghs et al., 2012; Ptak et al., 2012). Medina et al. (2008) found that left egocentric neglect was associated with hypoperfusion within right hemisphere dorsal stream areas while allocentric impairment was associated with damage to areas of the ventral visual processing stream. Similarly, Hillis et al. (2005) found that damage to the right superior temporal gyrus was implicated in allocentric neglect while damage to the right angular gyrus was predictive of egocentric neglect impairment. Chechlacz et al. (2012) conducted an anatomical likelihood estimate meta-analysis of 1306 neglect patients from 32 different lesion-symptom mapping studies which concluded that egocentric symptoms are associated with damage to the perisylvian network (e.g. pre- and postcentral gyrus, supramarginal gyrus, and superior temporal gyrus) while allocentric symptoms are associated with more posterior lesions impacting the angular, middle temporal, and middle occipital gyri. These findings support the characterisation of neglect as a cluster of distinct impairments rather than a unitary syndrome. However, these investigations focused exclusively on patients with left neglect following right hemisphere damage.

Few previous studies have investigated the neural correlates of visuospatial neglect following left hemisphere lesions. Suchan and Karnath (2011) conducted a lesion-symptom mapping analysis investigating the neural corelates of neglect following left hemisphere lesions. This investigation identified 11 patients with neglect and found that it was most strongly associated with damage to voxels within the left superior and middle temporal gyri, inferior parietal lobule, and insular cortex (Suchan and Karnath, 2011). Similarly, Beume et al. (2017) found that damage to the left superior and middle temporal gyrus, temporal pole, frontal operculum, and insula were the strongest predictors of right egocentric neglect within a cohort of 121 left hemisphere patients (21 with neglect). Additional studies have aimed to investigate the neural correlates of right egocentric neglect following left-hemisphere lesions, but these studies have employed categorical lesion comparisons (e.g. vascular territory, stroke severity) instead of more detailed voxel-wise analyses (Beis et al., 2004; Maeshima et al., 1992; Ogden, 1985; Ringman et al., 2004). Notably, no previous investigation has identified a statistically significant relationship between any brain area and right allocentric neglect impairment. Kleinman et al. (2007) investigated the anatomy of right allocentric neglect following left hemisphere damage. This study found that 2 of the 3 included patients exhibiting allocentric neglect in the absence of egocentric neglect had damage to Brodmann's areas 18, 19, and 37 in a descriptive overlay analysis (Kleinman et al., 2007). Overall, these findings demonstrate that allocentric neglect can occur following exclusively left hemisphere lesions, but the exact left hemisphere anatomy of this deficit has not yet been identified.

Suchan and Karnath (2011) propose that neglect occurs following damage to left hemisphere homologues to right-hemisphere spatial orienting areas. According to this theory, representation of spatial orienting in left hemisphere areas is thought to be a subdominant remnant of more primitive neural structure in humans. However, it is not yet clear whether this is an accurate characterisation of right neglect. Previous research has suggested that a small portion of right-lateralised egocentric neglect cases occur following ipsilesional right hemisphere lesions (4% as reported by (Agrell et al., 1997)). For example, Kim et al. (1999) assessed 30 right hemisphere stroke survivors on a line bisection task and identified 5 patients exhibiting ipsilesional (right) egocentric neglect deficits. This ipsilesional neglect could not be explained as a compensatory strategy, as these patients did not initially present with contralesional neglect impairment (Kim et al., 1999). Patients with ipsilesional neglect were found to have damage to right hemisphere fronto-subcortical circuits, suggesting that the right hemisphere may play a role in maintaining attention to ipsilesional hemispace. This finding is in line with the current understanding that the right hemisphere plays a role in distributing attention across both the right and left visual fields while the left hemisphere solely attends to contralesional hemispace (de Thiebaut de Schotten et al., 2011; Weintraub and Mesulam, 1987). However, it is not yet clear what implications this finding has concerning the overall anatomy of right egocentric neglect.

Most previous research investigating either right or left visuospatial neglect has systematically excluded patients exhibiting ipsilesional deficits and have only analysed the implicated anatomy within a single hemisphere (e.g. Chechlacz et al., 2010; Suchan and Karnath, 2011). Given that at least some cases of neglect have been found to ipsilesional, we suggest that is important to account for this heterogeneity by considering the full brain in lesion symptom mapping analyses within an unbiased cohort. The purpose of the present investigation is thus to conduct whole-brain voxel-based lesion symptom mapping analyses to identify the distinct neural correlates of left and right egocentric and allocentric neglect. Notably, the present investigation is the single largest whole brain lesion-mapping study to be conducted on visuospatial neglect as well as being the first to investigate the neural correlates of right allocentric neglect. Alongside conducting data-driven VLSM analyses, this study aims to investigate whether right-lateralised neglect is subserved by left hemisphere homologues of regions critical for left-lateralised neglect following right hemisphere damage.

## 2 Methods

### 2.1 Participants

This investigation considered existing data from a cohort of acute stroke survivors recruited as a component of the OCS-Tablet, OCS-Recovery, and OCS-Care studies (NHS REC reference 14/LO/0648, 18/SC/0550, and 12/WM/00335) (Demeyere et al., 2015, 2019). These studies recruited and examined a continuous sample of stroke survivors in 38 hospitals throughout the United Kingdom. Each included study employed limited exclusion criteria to only exclude patients who would not be able to concentrate for 10 min as judged by the multidisciplinary team. The patient recruitment was set to be as inclusive as possible for patients with aphasia, including witnessed consent and aphasia friendly testing with the OCS. We employed limited exclusion criteria to only exclude patients who would not be able to concentrate for 10 min as judged by the multidisciplinary team. The OCS was specifically designed to be inclusive for patients with aphasia (Demeyere et al., 2016; Mancuso et al., 2018), and indeed the sample included here shows high prevalence rates for language based tasks (32.4% impaired on the OCS picture naming task). Though severe comprehension deficits will still preclude testing with a cancellation task, Demeyere et al. (2015) showed that within a representative sample of 208 stroke survivors only 6.7% were unable to complete the cancellation task due to comprehension issues. This inclusion of aphasic patients is further evidenced by this study's lesion coverage (Fig. 2), which demonstrates sufficient overlap to facilitate testing within the left hemisphere regions most traditionally associated with aphasia. The OCS cancellation task has also been demonstrated to reliably assess neglect impairment, independent of interference from lateralised visual field deficits (Demeyere et al., 2015; Mancuso et al., 2018; Moore & Demeyere, Under Review).

All participants provided informed consent in accordance with the Declaration of Helsinki. Participants were included in this investigation if they had a confirmed diagnosis of acute stroke, were able to remain alert for 20 min, were at least 18 years of age, and were able to provide informed consent. Neuroimaging data was accessed from a database of clinical imaging from 1380 patients, of which 77.5% exhibited visible lesions. Participants were not pre-selected based on behavioural pathology or lesion location.

Cancellation data was available for 1100 acute patients of which 784 patients had available acute clinical imaging data. All patients with evidence of previous strokes (n = 99) or with total scores of less than 5 on the OCS Cancellation task (n = 21) were also excluded. Of the remaining patients, 194 had no visible stroke-related lesions and 24 scans demonstrated lesions which were unable to be normalised due to extensive atrophy or stroke-related midline shifts. A total of 446 patients (average age = 71.5 years (sd = 13.1), 44.5% female, 9.1% left handed) met all inclusion criteria for this investigation. This sample included 345 ischemic and 101 haemorrhagic stroke patients (224 R, 191 L, 31 Bilateral). The average stroke-test interval was 5.85 days (sd = 5.99) with 76.8% of patients completing behavioural testing within the first week following stroke. Similarly, the average stroke-scan interval was 1.67 days (sd = 3.73) with 93.9% of patients completing their relevant clinical scan within the first week following stroke. See Table 1 for a detailed breakdown of patient clinical characteristics. All patients classed as “bilateral” exhibit evidence of a single, spatially-contiguous lesion which partially crosses the midline. These patients are explicitly included in this investigation, as excluding patients with these damage patterns would preclude the identification of any significant neural correlates within similar regions which are most frequently impacted by strokes which cross the anatomical midline. For example, many pontine and large cerebellar strokes cross the midline due to the vascular anatomy of these regions. For this reason, patients were not pre-selected based on stroke location.

**Table 1:** A breakdown of the clinical characteristics of patients within each VLSM analysis group. Test Date reports the interval between stroke and behavioural testing in days. Scan Date reports the interval between stroke and neuroimaging data collection in days. Lesion sizes are reported in cm^3^. Standard deviations are provided in parentheses. Visual field deficits as classified by the OCS are provided. L = Left, R = Right, B = Bilateral, Ego = Egocentric, Allo = Allocentric, VFD = Visual Field Deficit.

	Demographics						Lesion Details				Behaviour			
	N	Age	Female	L Handed	Test Data	Scan Date	Size	L	R	B	Total	Ego Score	Allo Score	VFD
**Left Ego**	58	72.9 (11.6)	55.2%	15.4%	6.2 (6.5)	1.4 (3.24)	5.9 (5.8)	6	48	4	26.8 (11.4)	0.72 (0.56)	0 (0.03)	8 L, 3 R
**Left Allo**	27	72.0 (13.8)	48.1%	10.5%	4.5 (6.2)	2.6 (4.0)	5.5 (7.2)	8	17	2	32.4 (14.5)	0 (0.1)	0.2 (0.2)	3 L, 1 R
**Left Ego & Allo**	42	74.5 (10.9)	38.1%	0.0%	5.6 (5.7)	1.4 (2.3)	10.9 (11.1)	2	39	1	17.4 (11.3)	1.30 (0.63)	0.48 (.30)	14 L, 1 R
**Right Ego**	31	72.5 (10.6)	51.6%	13.3%	5.9 (5.7)	1.1 (1.7)	3.39 (43.2)	22	8	1	31.8 (10.4)	−0.50 (0.12)	0.02 (0.04)	0 L, 6 R
**Right Allo**	28	73.6 (13.7)	46.4%	18.8%	5.4 (4.2)	3.2 (5.1)	5.93 (9.02)	19	7	2	36.4 (13.6)	−0.03 (0.12)	−0.11 (0.09)	0 L, 4 R
**Right Ego & Allo**	11	78.7 (7.3)	63.6%	0.0%	5.1 (5.4)	2.4 (3.0)	4.3 (7.1)	5	4	2	24.0 (10.6)	−0.91 (0.63)	−0.21 (0.12)	2 L, 1 R
**No Neglect**	249	69.8 (14.0)	40.0%	8.3%	6.0 (6.1)	1.6 (4.1)	3.0 (4.5)	101	129	19	40.6 (10.6)	0.00 (0.11)	0.00 (0.03)	6 L, 11 R

### 2.2 Behavioural data

**Fig. 1:**
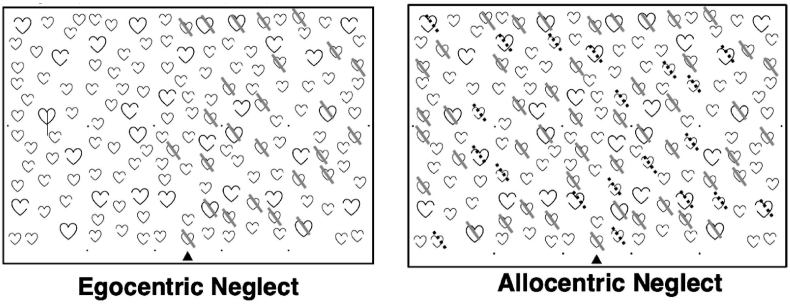
An example of egocentric/allocentric neglect impairment as captured by the Oxford Cognitive Screen's Hearts Cancellation Task. Patients with egocentric neglect omit targets on one side of the page while patients with allocentric neglect commit consistently lateralised false positive errors.

Data from the OCS Hearts Cancellation Task was considered in this investigation. The Hearts Cancellation Task is a standard neuropsychological assessment designed to detect and differentiate between egocentric and visuospatial neglect impairments (Fig. 1). This test has been demonstrated to be highly sensitive to neglect impairment (94.12%, versus the Behavioural Inattention Test Star Cancellation – Demeyere et al. (2015)). In this task, patients are presented with line drawings of 150 hearts (50 complete, 50 left-gap, 50 right-gap) pseudorandomly scattered across an A4 sheet of paper (Fig. 1). Patients are instructed to cross off all complete hearts while ignoring incomplete distractor stimuli and are allowed 3 min to complete this task.

**Fig. 2:**
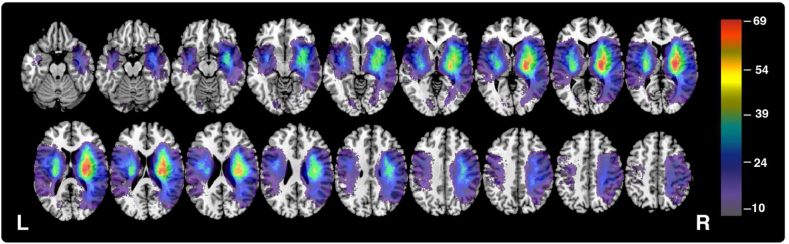
Lesion overlay for the total sample of 446 participants. Colour represents number of patient lesions overlapping within each region. Only regions with a minimum overlap of 10 are visualised (MNI z coordinates -44 – 66). The voxels highlighted in this visualisation are tested in all reported VLSM analyses. (For interpretation of the references to colour in this figure legend, the reader is referred to the Web version of this article.)

The terms left/right egocentric neglect refer to patients who commit left-lateralised or right lateralised target omission errors respectively. Similarly, the terms left/right allocentric neglect refer to patients who commit left-lateralised false positive errors and right-lateralised false positive errors respectively. According to normative OCS impairment thresholds, egocentric scores greater than 3 or less than −3 and allocentric scores greater than 1 or less than −1 represent significant impairment (Demeyere et al., 2015). These standard thresholds were used to binarize impairment categorisations (e.g. Table 1). However, more fine-grained continuous measures were used to quantify impairment severity for VLSM analysis. In the VLSM analyses, egocentric severity was quantified using a centre of cancellation measure (Rorden and Karnath, 2010). This metric was calculated by assigning each target a numeric weight based on location along the right/left axis, and averaging these weights (see Huygelier et al., 2020; Moore et al., 2021). Similarly, allocentric asymmetry was quantified by dividing the number of consistently-lateralised false positive responses by the number of reported targets (see Moore et al. (2021).

### 2.3 Lesion data

The extent and location of patient lesions was quantified using clinical CT (n = 376) and MR (61 T2, 3 T1, 6 FLAIR) whole-brain scans obtained as a component of routine post-stroke clinical imaging. Patient lesions were manually delineated on native space scans using MRIcron (McCausland Centre for Brain Imaging, Columbia, SC, USA) by investigators who were blind to behavioural results (Varjacic et al., 2018). All lesion masks were smoothed at 5 mm full width at half maximum in the z-direction and binarized using a 0.5 threshold using built-in MRIcron smoothing functions. Smoothing is a standard lesion pre-processing step which helps prevent minor variations in delineation user input from impacting analysis (de Haan and Karnath, 2018). These scans and lesion masks were then reoriented to the anterior commissure and warped into 1 × 1 × 1 mm stereotaxic space using the Statistical Parametric Mapping 12 and Clinical Toolbox (Rorden et al., 2012) functions. All normalised scans and lesions were visually inspected for quality before conducting lesion mapping analyses. This lesion preparation process represents a standard analysis pathway which has been used in a number of previous VLSM investigations (e.g. Varjačić et al., 2018).

### 2.4 Analysis

Four VLSM analyses were conducted to determine the neural correlates egocentric and allocentric neglect within patients exhibiting left and right visuospatial neglect. These VLSM analyses were conducted on a theory-blind voxel-wise basis using the MATLAB package NiiStat (https://github.com/neurolabusc/NiiStat). Given that continuous impairment severity metrics (centre of cancellation or allocentric proportional scores) were employed, this software employed one-tailed pooled-variance t-tests to evaluate voxel significance. Only voxels which were lesioned in a minimum of 10 patients were considered (n = 589,216, Fig. 2). These analyses employed a highly conservative Bonferroni correction (corrected alpha = 8.49 × 10^−8^, z-cut = 5.23) and controlled for lesion volume. This conservative analysis approach was employed to harness this study's extremely large sample size and testing space to prioritize specificity over sensitivity (Sperber and Karnath, 2017). Specifically, this analysis aims to locate “core”, highly significant lesion sites rather than peripheral areas which are less strongly associated with neglect impairment. Lesion anatomy was evaluated versus the Harvard-Oxford Cortical (Desikan et al., 2006) and John's Hopkins University White Matter (Mori et al., 2005; Wakana et al., 2007) atlases.

Finally, the voxel maps produced by each VLSM analysis were compared to analyse the degree of overlap between voxels associated with different neglect impairments. Specifically, voxel maps for similarly-lateralised egocentric and allocentric neglect were compared to confirm whether these conditions can be dissociated at an anatomical level. Next, the voxel maps associated with left hemisphere neglect impairments were inverted to overlay with their right-hemisphere homologues in order to quantify the degree of similarity between the correlates of right and left hemisphere neglect. All analysis output files and behavioural data are openly available on the Open Science Framework (Foster and Deardorff, 2017) (https://osf.io/vf9ew/). All additional data is available upon request.

## 3 Results

**Fig. 3:**
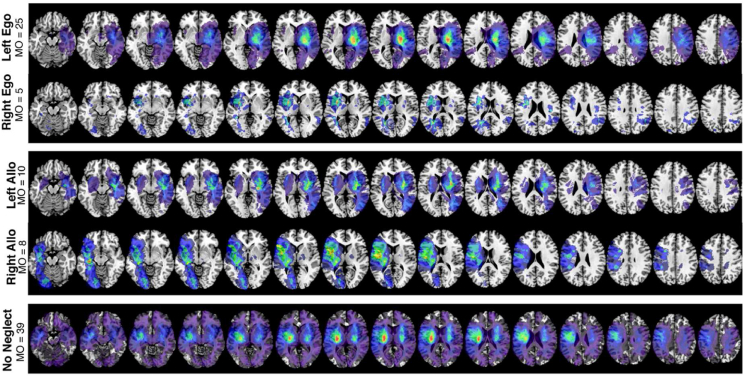
Lesion overlays each of the neglect impairment subgroups. Only lesions from impaired patients are visualised. Colour indicates number of lesion overlaps present within each region (minimum overlap = 1). MNI z coordinates -44 – 66 are visualised. MO = maximum overlap (represented in red). Notably, these overlays should not be interpreted as an overlap analysis due to confounds associated with stroke arterial territories (Mah et al., 2014). Instead this figure is provided to illustrate the analysis sampling space areas which are able to be analysed for each neglect subtype. (For interpretation of the references to colour in this figure legend, the reader is referred to the Web version of this article.)

Fig. 2 presents the lesion overlay for all 446 participants within the voxels included in VLSM analysis. The highest lesion overlap (n = 69) was present within the MCA territory. Patients with neglect (defined as any impairment on the ego and/or allocentric measures, n = 197) were found to have significantly larger lesions than participants without neglect (mean volume 51.57 cm^3^ versus 24.12 respectively, t (445) = 14.793, p < 0.001). A one-way ANOVA analysis revealed a significant relationship between neglect type and lesion volume (F (5,191) = 4.026, p = 0.002). Post-hoc Tukey HSD tests revealed that this effect was driven by a significant difference in severity between patients with left egocentric and both left neglect types (47.4 vs 87.3, p = 0.0224) and a significant difference in volume between patients with right egocentric and both left impairments (27.2 vs 87.3, p < 0.001). All other conducted volume comparisons were not significant. Notably, 35/197 (17.8%) of included neglect patients demonstrated ipsilesional neglect impairment. Fig. 3 presents descriptive lesion overlays of patients in each of these behavioural impairment categories.

### 3.1 VLSM analysis

**Fig. 4:**
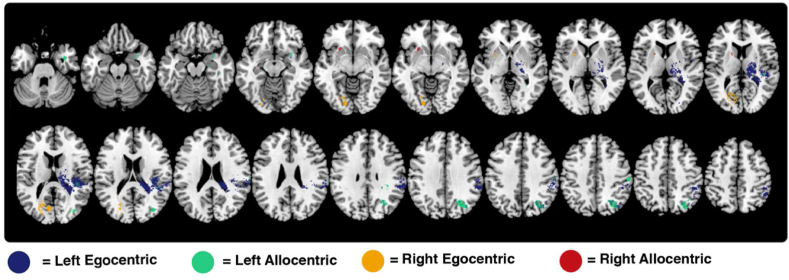
A visualisation of the voxels surviving highly conservative Bonferroni correction for each VLSM analysis. Horizontal slices between MNI z coordinates -44 –66 are visualised.

**Fig. 5:**
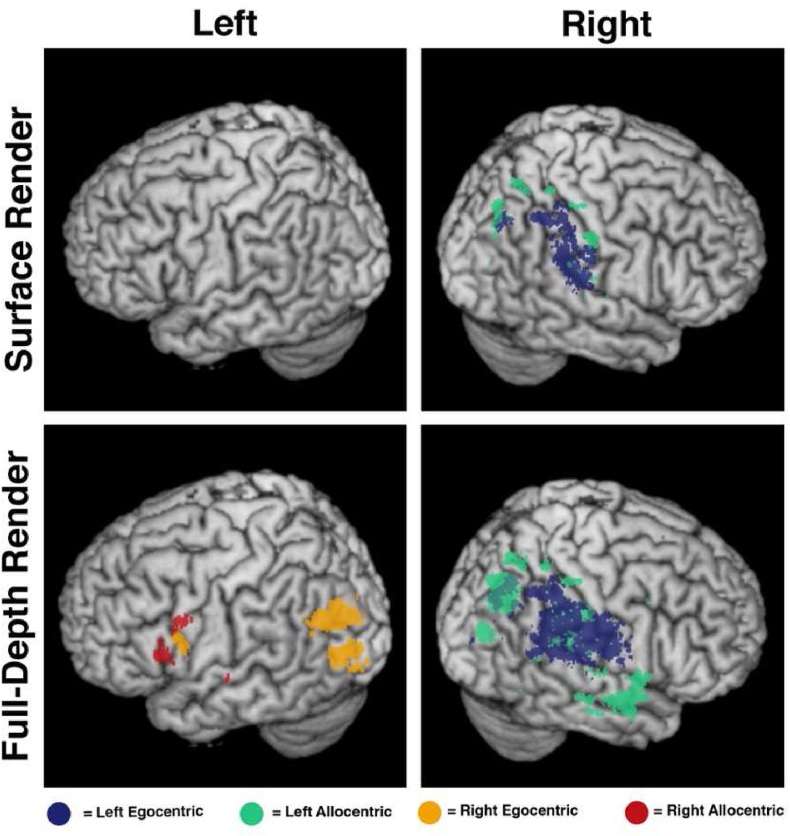
A 3D render of significant voxels for each conducted comparison. The surface render visualises all voxels within 4 voxels of the brain surface. The full-depth render visualises all significant voxels, regardless of depth. Importantly, this depth render should not be considered in isolation, but should be compared to the axial slices presented within Fig. 4 to provide a detailed visualisation of the significant voxels within 3D space.

First, two VLSM analysis were conducted to identify the neural correlates of left egocentric and left allocentric neglect (Table 2, Fig. 4, Fig. 5). VLSM analysis of left egocentric neglect yielded 11,526 significant voxels with the peak z-score (z = 7.392) centred within the left parietal operculum (MNI 39–34 19). This significant voxel cluster impacted a number of left temporo-pareital cortical areas including the supramarginal gyrus, and planum temporale, lateral occipital cortex (superior division). Voxels within underlying white mater tracts including the internal capsule (posterior and retrolenticular parts), tapetum, and posterior corona radiata were also significantly associated with left egocentric neglect impairment. Full anatomical statistics of significant voxels are reported in Table 2.

**Table 2:** Detailed anatomical descriptions of the significant voxel clusters identified in each individual VLSM analysis. Starred ROIs contain the peak z-values for each VLSM test. Full anatomical descriptions for each voxel cluster are available on the Open Science Framework. Fraction represents the proportion of each ROI covered by each significant z-statistic map. All anatomical areas are defined based on the Harvard-Oxford Cortical Atlas (HAROX) and the Johns Hopkins University White Matter Atlas (JHU). Hem = hemisphere (Left/Right). Nsig = number of significant voxels within each ROI.

Left Egocentric		Hem.		Nsig	Fraction	Atlas
Supramarginal Gyrus (Posterior Division)		R	1698		11.37%	HAROX
Planum Temporale		R	1087		20.43%	HAROX
Parietal Operculum Cortex*		R	972		17.94%	HAROX
Lateral Occipital Cortex (Superior Division)		R	946		2.01%	HAROX
Supramarginal Gyrus (Anterior Division)		R	696		8.77%	HAROX
Internal Capsule (Posterior Limb)		R	633		13.62%	JHU
Tapetum		R	552		62.80%	JHU
Internal Capsule (Retrolenticular Part)		R	408		12.42%	JHU
Heschl's Gyrus		R	257		7.90%	HAROX
Corona Radiata (Posterior)		R	237		4.69%	JHU
Angular Gyrus		R	209		1.44%	HAROX
Splenium of Corpus Callosum		R	192		1.21%	JHU
Posterior thalamic Radiation		R	173		3.33%	JHU
Superior Longitudinal Fasciculus		R	74		0.78%	JHU
Insular Cortex		R	48		0.42%	HAROX
Central Opercular Cortex		R	30		0.35%	HAROX
Superior Temporal Gyrus (Posterior Division)		R	29		0.29%	HAROX
Fornix (cres) Stria terminalis		R	23		1.22%	JHU
External capsule		R	18		0.22%	JHU
Body of Corpus Callosum		R	11		0.07%	JHU
**Left Allocentric**						
Lateral Occipital Cortex (Superior Division)*	R		2589		5.50%	HAROX
Supramarginal Gyrus (Anterior Division)	R		189		2.38%	HAROX
Planum Temporale	R		157		2.95%	HAROX
Superior Parietal Lobule	R		101		0.63%	HAROX
Temporal Pole	R		74		0.31%	HAROX
Postcentral Gyrus	R		63		0.19%	HAROX
Uncinate Fasciculus	R		63		10.23%	JHU
Supramarginal Gyrus (Posterior Division)	R		55		0.37%	HAROX
Superior Longitudinal Fasciculus	R		41		0.43%	JHU
Parietal Operculum Cortex	R		28		0.52%	HAROX
Temporal Fusiform Cortex (Posterior Division)	R		28		0.35%	HAROX
Precuneous Cortex	R		26		0.10%	HAROX
Inferior Temporal Gyrus (Posterior Division)	R		25		0.21%	HAROX
Insular Cortex	R		20		0.17%	HAROX
Parahippocampal Gyrus (Anterior Division)	R		19		0.23%	HAROX
Middle Temporal Gyrus (Posterior Division)	R		18		0.14%	HAROX
External Capsule	R		15		0.19%	JHU
Planum Polare	R		11		0.25%	HAROX
**Right Egocentric**						
Intracalcarine Cortex	L		381		5.50%	HAROX
Occipital Fusiform Gyrus*	L		275		2.22%	HAROX
Lingual Gyrus	L		271		1.72%	HAROX
Supracalcarine Cortex	L		186		5.60%	HAROX
Lateral Occipital Cortex (Superior Division)	L		91		0.20%	HAROX
Posterior Thalamic Radiation	L		43		0.80%	JHU
Splenium of Corpus Callosum	L		24		0.15%	JHU
Cuneal Cortex	L		17		0.28%	HAROX
**Right Allocentric**						
External capsule	L		118		1.46%	JHU
Internal Capsule (Anterior Limb)*	L		56		1.40%	JHU

Conversely, VLSM analysis of right egocentric neglect yielded 1556 significant voxels with the peak z-score (z = 7.151) located within the left occipital fusiform gyrus (MNI -17 -74 37). Significant voxels were organised in two distinct clusters. The posterior cluster impacted occipital cortical areas including the intercalcarine cortex, lingual gyrus, and supracalcarine cortex. Voxels within the left posterior thalamic radiation and corpus callosum (splenium) were also significantly associated with right egocentric neglect impairment. The more anterior cluster impacted subcortical areas underlying the left insular cortex, primarily the anterior putamen.

To evaluate anatomical overlap between left and right egocentric neglect, the left hemisphere voxels which were found to be significantly associated with right neglect were superimposed on the homologous voxels within the right hemisphere. Only one voxel of overlap was present between the voxels associated with left egocentric neglect and the right hemisphere homologues of voxels associated with right egocentric neglect impairment. This voxel was centred near the inferior medial border of the lateral occipital cortex (superior division).

VLSM analysis of left allocentric neglect impairment yielded 3968 significant voxels with a peak z-score of 7.150 centred within the superior division of the right lateral occipital cortex (MNI 25–65 59). The significant voxels were largely grouped in three clusters. First, a cluster of significant voxels was present within the lateral occipital cortex. This cluster partially overlapped with voxels associated with left egocentric neglect, but included a number of distinct, more inferior voxels which were not found to be associated with left egocentric impairment. Next, a cluster of significant voxels was centred on the border between the post-central gyrus and the anterior division of the supramarginal gyrus. A third, more anterior cluster of significant voxels was located on the inferior border between the planum temporale, temporal pole, posterior temporal fusiform cortex, and anterior parahippocampal gyrus. Overall, 6.45% of significant voxels were associated with both left egocentric and left allocentric neglect. These overlapping voxels were centred within the lateral occipital cortex (superior division) and planum temporale.

Finally, VLSM analysis of right allocentric neglect yielded 203 significant voxels with a peak z value (z = 5.949) located within the anterior limb of the left internal capsule. This cluster impacted the external capsule. In total, there was 0.34% overlap between the voxels significantly associated with right allocentric and right egocentric neglect. These 6 overlapping voxels were located within the white matter underlying the right insular cortex. The left hemisphere voxels associated with right allocentric neglect were then superimposed on their right hemisphere homologous to evaluate the anatomical overlap between left and right allocentric neglect. No common voxels were present within this comparison.

## 4 Discussion

The purpose of the present study was to identify the neural correlates of left and right lateralised visuospatial neglect in a large, representative sample of acute stroke survivors and to determine whether right lateralised neglect is subserved by homologous areas underlying left-lateralised neglect following right hemisphere damage. As in previous studies, left egocentric neglect was found to be most strongly associated with damage to the right temporoparietal area while left allocentric neglect was associated with distinct, more posterior and inferior (or ventral) lesion sites. Conversely, right egocentric neglect was found to be most strongly associated with damage to left hemisphere occipital areas while right allocentric neglect was related to damage to voxels within the left internal capsule white matter. While there was some degree of overlap (6.45%) between the neural correlates associated with left egocentric and left allocentric neglect, the voxels associated with right egocentric and right allocentric neglect demonstrated less than 0.5% overlap. The right hemisphere homologues of the regions associated with right allocentric neglect did not overlap with the regions associated with left allocentric neglect. Similarly, only one voxel of overlap was present between the regions associated with left egocentric neglect and the right hemisphere homologues of voxels associated with right egocentric neglect impairment. These findings provide important novel insights into the neural correlates of spatial attention.

The behavioural data collected in this investigation provides a clear demonstration of heterogeneity within the neglect syndrome. Within the consecutive sample included in this investigation 28.5% exhibited right-lateralised and 15.7% exhibited left-lateralised neglect. This finding is in line with previous research demonstrating that neglect deficits regularly occur following both right and left hemisphere damage (Moore et al., 2021; Stone et al., 1992; Ten Brink et al., 2017). Similarly, as in previous studies, egocentric and allocentric neglect were found to be behaviourally doubly dissociated (Chechlacz et al., 2010, 2012). Notably, 17.8% of the neglect patients identified in this investigation exhibited ipsilesional neglect deficits. Previous VLSM studies have generally included a specific subset of neglect patients, rather than a broad, representative sample. For example, may previous investigations have excluded all left hemisphere or ipsilesional neglect patients, and have not distinguished between egocentric and allocentric neglect impairment (Chechlacz et al., 2012; Molenberghs et al., 2012; Suchan and Karnath, 2011). It is critically important to adequately represent this variety within experimental subsets in order to produce generalisable results which can further fundamental understanding of the neglect syndrome as a whole.

Left egocentric neglect was primarily associated with damage within the right temporo-parietal cortex and underlying white matter while left allocentric neglect was found to be associated with distinct voxel clusters. These distinct clusters were centred within the left lateral occipital cortex and the inferior border of the anterior parahippocampal gyrus. A third cluster of significant voxels was present on the border between the post-central gyrus and the anterior division of the supramarginal gyrus. This third cluster largely overlapped with voxels found to be significantly associated with left egocentric neglect impairment. Notably, significant voxels were present within the right lateral ventricle. This finding is likely due to the inclusion of patients with haemorrhages and ischemic-related swelling causing lesions to space normally occupied by the lateral ventricles (Lebby, 2013). These findings are in line with previous findings suggesting that left egocentric and allocentric neglect are associated with distinct but overlapping regions. Some degree of this overlap may be accounted for by the common co-occurrence of left egocentric and allocentric neglect, with 42 (9.4%) patients included in these analyses exhibiting both impairments.

Conversely, right egocentric neglect was most strongly predicted by damage to left hemisphere occipital areas including the intercalcarine, lingual, and occipital fusiform cortices. These results contrast with previously reported results which have identified correlates of right egocentric neglect within left frontotemporal cortical areas. However, this disagreement can be largely accounted for by the differences in methodologies employed by these studies. First, previous investigations have restricted analyses to the left hemisphere, precluding detection of any right hemisphere correlates (Beume et al., 2017; Suchan and Karnath, 2011). Second, Suchan and Karnath (2011) did not explicitly distinguish between egocentric and allocentric neglect. This investigation quantified allocentric-level biases within a figure copy task, but grouped patients into “neglect” and “no neglect” VLSM analysis groups regardless of the type of neglect impairment exhibited. This “neglect” group may have then included a combination of patients with pure egocentric, pure allocentric, and both egocentric and allocentric neglect. Previous research has established that left egocentric and allocentric neglect are associated with distinct neural correlates. It therefore seems likely that similar variation exists within right neglect, meaning that failing to distinguish between egocentric and allocentric neglect may likely introduce confounding noise into anatomical analyses.

Notably, right egocentric neglect was associated with damage to areas which are traditionally associated with lateralised visual rather than attentional impairments. Specifically, voxels within the primary visual cortex (intercalcarine cortex) were found to be associated with right egocentric neglect impairment. The OCS cancellation task reliably distinguishes between neglect and low-level visual impairments, as patients with visual impairment retain the ability to visually scan the search matrix whilst patients with neglect do not (Demeyere et al., 2015). Previous research has demonstrated that occipital areas, including those found to be associated with right egocentric neglect, may play a key role in directing visual attention (Vilares et al., 2012). For example, Vilares et al. (2012) found that a cluster of voxels centred within the superior occipital cortex and extending into the calcarine sulcus and lingual gyrus was responsible for encoding salience-based aspects of visual attention. These regions were also found to be associated with right egocentric neglect, implying a degree of functional overlap. Alternatively, it is possible that the association between the intercalcarine cortex and egocentric neglect is indicative of a general association between PCA strokes and egocentric neglect. There is a degree of debate surrounding whether patients with visual field deficits or exclusively occipital lesions should be included in lesion mapping analyses aiming to identify the correlates of neglect (Ten Brink et al., 2019). However, this study aims to accurately represent the anatomical heterogeneity present within the stroke population and excluding these patients would remove a key, underrepresented patient group from this analysis (Bird, 2006; Mort et al., 2003). Additionally, it has been demonstrated that the visual deficits associated with isolated occipital lesions do not cause neglect deficits and are therefore unlikely to bias the results of the conducted analysis (Park et al., 2006). Additional research is needed to determine the exact mechanisms underlying right egocentric neglect in order to further understand the functional role of these posterior lesion sites.

Previous research investigating the neural correlates of right egocentric neglect has generally concluded that this impairment is associated with damage to more anterior temporo-parietal areas rather than the occipital areas identified in this investigation (Beume et al., 2017; Suchan and Karnath, 2011). This discrepancy can be largely accounted for by methodological differences. First, previous analyses have considered a restricted sample of neglect patients (n = 11, n = 21) with exclusively left hemisphere damage while the present study conducted whole-brain analyses on a much larger sample of right egocentric neglect patients (n = 42). In previous studies, the vast majority of patients have exhibited MCA territory strokes, with limited overlap present in the regions found to be associated with neglect in the present study (Beume et al., 2017; Suchan and Karnath, 2011). This is in line with the clinical picture of the majority of patients exhibiting MCA damage, but precludes testing the role of more posterior lesion sites in right egocentric neglect impairment. However, it necessary to include a larger sample with more diverse lesions in order to gain a more accurate understanding of right egocentric neglect.

Finally, the present study explicitly distinguished between egocentric and allocentric neglect impairment. Suchan and Karnath (2011) did include behavioural tests of allocentric-level impairment, but did not conduct VLSM analyses which distinguished between egocentric and allocentric neglect. Given that right allocentric neglect was found to be associated with more anterior fronto-temporal regions, failing to exclude these patients from an analysis of right egocentric neglect would be expected to bias results towards these anterior areas. This is due to the fact that VLSM is most effectively able to identify the correlates of functions which are associated with a single, spatially contiguous correlate and tends to “average” across spatially distinct correlates (Bates et al., 2003; de Haan and Karnath, 2018). This “averaging” effect is particularly prominent when small samples of impaired patients are used (de Haan and Karnath, 2018). In the present study, this effect was carefully controlled for by regressing out lesion volume and employing an extremely conservative correction for multiple comparisons.

Overall, the findings of this experiment strongly suggest that right egocentric neglect cannot be accurately conceptualised as resulting from damage to left-hemisphere homologues of traditional right spatial attention areas. Damage to right hemisphere homologues of the left hemisphere voxels implicated in right egocentric was not found to be associated with left egocentric impairment. Some degree of this disagreement can be accounted for by differences in the VLSM analyses, given that both analyses included a different lesion overlay patterns (Fig. 2). This diversity implies that even if right and left egocentric were exactly homologous, some degree of disagreement would be present in the VLSM results. However, this inherent noise would not be expected to result in the clear, qualitative differences between right and left egocentric neglect documented in this investigation. Left egocentric neglect was largely associated with damage to temporo-parietal areas while right egocentric impairment was associated with posterior occipital regions. This diversity implies that egocentric neglect is best understood as a common symptom of multiple underlying lesion patterns, rather than a unitary syndrome. It is important for future research to take this anatomical and behavioural diversity into account in order to produce findings which can help further fundamental understanding of the neglect syndrome as a whole. Additionally, future research is needed in order to determine how right-lateralised egocentric neglect mechanistically differs from left-lateralised neglect deficits.

Notably, the present study is the first to conduct statistical analyses aiming to identify the neural correlates of right allocentric neglect. This deficit was found to be significantly associated with a small cluster of voxels centred within right internal capsule. This finding differs from the previously reported results of Kleinman et al. (2007). This discrepancy is likely due to the present study's comparatively larger sample of allocentric neglect patients (39 patients versus 3 patients) and use of quantitative statistical analyses rather than qualitative lesion descriptions. Inferior temporo-frontal cortical regions have been consistently associated with object-level perceptual processes and is a key anatomical component of the ventral visual processing stream (Borowsky et al., 2007; Grill-Spector, 2003; Quiroga et al., 2005). It is plausible that damage to the external and internal capsule white matter tracts disrupts communication with these areas, resulting in allocentric neglect impairment. However, there was no overlap between the neural correlates of left and right allocentric neglect. This suggests that the allocentric level spatial-attentional system is not homologous across hemispheres. Additional research is therefore needed to provide a more detailed characterisation of the neural correlates of allocentric-level visual attention in order to better understand the exact mechanisms which underly this condition.

Considered cumulatively, the results of the present investigation support the characterisation of visuospatial neglect as a heterogenous cluster of impairments rather than a unitary syndrome. Similarly, the consistent involvement of damage to white matter tracts in egocentric/allocentric neglect supports previous assertions that neglect represents a disconnection syndrome in which neglect symptoms with distinct cortical correlates may be linked back to common white matter damage. Future investigations need to account for this heterogeneity. Studies which do not account for differences between left/right or egocentric/allocentric neglect deficits may risk producing overgeneralised results confounded by considering distinct behavioural deficits as a single, unitary impairment. The inclusion of large, representative patient samples assessed for both egocentric and allocentric level neglect impairment is critical in order to facilitate valid conclusions about the neglect syndrome as a whole.

### 4.1 Limitations

This investigation exclusively employed routinely collected clinical neuroimaging and behavioural data (84.3% CT, 15.7% MRI). While this approach allowed for the inclusion of the largest number of patients in any neglect VLSM study, this methodology is not without limitations. Acute lesions develop over time and are often not fully visible on acute CT imaging (González, 2005; Merino and Warach, 2010). Ideally, more high-resolution imaging data from T2 MRI or DWI scans would be employed to quantify lesion anatomy. Given that this investigation employed routinely collected clinical imaging, these higher quality scans were only available for a subset of the included patients. However, previous investigations have repeatedly demonstrated the feasibility of employing acute CT scan data for VLSM analysis (de Haan and Karnath, 2018; Varjačić et al., 2018). Regardless of imaging modality, there is inherently some degree of measurement error present within creating binarized, quantitative lesion masks from uncertain clinical data. We have to minimised the impact of this error by including only lesion masks which were confirmed by trained researchers and by using extremely strict Bonferroni corrections when conducting VLSM analysis.

Post-stroke diaschisis and/or hypoperfusion effects can potentially introduce noise into studies using acute imaging to quantify brain-behaviour relationships, as post-stroke impairments are not only caused by localised tissue damage but also involve altered functional connectivity between intact regions (Gillebert and Mantini, 2013; Karnath et al., 2011). Similarly, non-random spatial distributions due to arterial territories can potentially distort lesion-mapping results (Mah et al., 2014). These issues are inherent within traditional VLSM analyses and future research could aim to employ network-based, multivariate lesion mapping approaches in order to understand how these effects can impact results.

Previous research has suggested that it is generally best to diagnose neglect based on agreement between multiple or repeated neuropsychological tests as no single neuropsychological test is perfectly sensitive to neglect impairment (Azouvi et al., 2002; Huygelier et al., 2020; Lindell et al., 2007). Though additional and potentially computerised measures may have provided a more sensitive measure, the acute nature and relatively severe neglect impairments combined with a sensitive cancellation task provides confidence in the methods employed. However, given this investigation represents a retrospective analysis of routinely collected data, no additional neglect behavioural assessment data were available.

VLSM is inherently better at identifying the neural correlates of deficits which are subserved by a single, spatially coherent neural correlate (Gajardo-Vidal et al., 2018). Given that visuospatial neglect deficits likely involve disconnection syndromes rather than a single critical lesion site, additional research using a range of analysis techniques is needed to further clarify the underlying networks implicated in spatial attentional deficits. In addition to impacting different spatial reference frames, Patients with visuospatial neglect can exhibit a diverse range of location or modality-specific behavioural phenotypes and functional outcomes (Aimola et al., 2012; Binder et al., 1992; Connine et al., 1990; Guilbert et al., 2016; Laplane and Degos, 1983; Moore et al., 2021; Ten Brink et al., 2019). Future research is needed to specifically identify the neural correlates of these additional neglect subtypes.

### 4.2 Conclusion

The findings of this investigation elucidate the distinct neural correlates of right and left visuospatial neglect deficits in a large, representative sample of stroke survivors. The findings pertaining to left egocentric/allocentric neglect align with and replicate findings from existing literature. With regards to the critical neuro-anatomy underlying right-lateralised visuospatial neglect, where little research exists, we found right egocentric neglect to be most strongly predicted by damage to voxels within the posterior occipital cortex. Importantly, right neglect should not be characterised as a consequence of damage to left-hemisphere homologues of the right hemisphere attentional systems, but instead may represent a common behavioural consequence of an anatomically diverse range of underlying lesions. Notably, this is the first study to identify the neural correlates of right allocentric neglect in the left internal/external capsule white matter. Cumulatively, these findings provide novel insight into the neural correlates of spatial attention.

## Author contributions

MJM conducted analysis and drafted the manuscript. CG supervised VLSM analysis, aided in study conceptualisation, and edited manuscript drafts. ND aided in study conceptualisation and edited manuscript drafts.

## Funding

This work was funded by 10.13039/501100000364Stroke Association UK awards to ND (TSA2015_LECT02; TSA, 2011/02) and MJM (SA PGF 18\100,031), the 10.13039/100004440Wellcome Trust award to CRG (101253/A/13/Z) and was supported by the 10.13039/501100000272National Institute for Health Research (NIHR) Oxford 10.13039/100014461Biomedical Research Centre (BRC) based at 10.13039/501100006149Oxford University Hospitals NHS Trust.

## Declaration of competing interest

None.
